# Louisville Surgical Society

**Published:** 1894-09

**Authors:** 


### Society Proceedings.

### LOUISVILLE SURGICAL SOCIETY.

*Stated Meeting, February, 1894.*

Dr. Ap. M. Vance, president, in the chair.

Dr. I. N. Bloom (Urethritis; Simple and Specific): I have recently had two or three cases which have furnished considerable food for reflection. They were all cases of urethritis. It is now a generally accepted fact, among those who are supposed to know, that urethritis is divided into three classes: (1) Traumatic urethritis; (2) urethritis simplex; (3) urethritis specific.

A man having gonorrhea is subjected to specific urethritis, which is brought about by the specific germ, the gonococcus of Neisser, carried from one subject to another, and by its development produces the disease. While there have been some differences of opinion in regard to the causation of gonorrhea, the above is the most generally accepted theory. I am not prepared to dispute this, but two cases I wish to report have, at least, been food for reflection for me for some time, and I propose to give the cases first, then draw my deductions therefrom, or leave the members of the society to draw theirs.

The first case came under my observation about a year and a half ago. A man who had been married about ten years contracted gonorrhea, at least gonorrhea was the diagnosis given by his physician. The following are the particulars: The man had been on a spree, and had indulged in one sexual intercourse away from his wife. Two or three days after this, having no symptoms of any kind, he had connection with his wife. About a day following this coitus he noticed a slight discharge from his penis, and consulted me. The flow, when I saw it, was mucoid, scarcely muco-purulent, in character, about the color and consistency of glycerine. Urination on the part of the patient was painless, and there was little or no inflammation around the meatus. At the end of four or five days of ordinary treatment there was no discharge at all from the penis or in the urine ; in the meantime, however, for one or two days, the flow became more purulent in character, but principally mucoid, and remained that way until probably the end of five days, when it ceased altogether. He had not practised coitus with his wife from the time he first consulted me, and she subsequently developed undoubted gonorrhea, which lasted four or five weeks, severe in character, with urethritis, vaginitis and pelvic peritonitis, characterized by the usual symptoms and usual amount of severe pain. Previous to the time this man first consulted me he had no signs of gonorrhea for ten years.

The other case (rather there are two of them which are practically alike, one of which I followed very closely, having had a microscopical examination of the discharge made by Dr. Vissman,) occurred in a young man, twenty-six years of age, who came to me about a year and a half ago for a syphilitic affection which was then about two years old. About the same time he told me of a gonorrheal discharge he had, which came on at intervals ; sometimes for a week or two it would remain quiet, then become varied in form from a thin, gleety, mucous discharge to (at times) a decidedly purulent flow. In the course of treatment for syphilis I saw the patient quite frequently, and he paid little or no attention to this discharge; in fact, the urethral discharge was only mentioned occasionally, and received no direct or thorough treatment. The patient seemed disinclined to it, would not use injections nor internal medicines faithfully, and would not limit his desires or the gratification of them.

In June the man fell in love and began to think seriously of marriage. In the meantime three years had elapsed since the development of syphilitic manifestations, to which he had attended fairly faithfully as a man can do by getting a month's supply of medicine and taking it regularly. His gonorrhea, from his failure to give the treatment of it proper attention, had become chronic. About the last of July, when he consulted me as to the possibility of marriage, I told him that so far as syphilis was concerned there was no reason why he should not marry, but so long as there was the slightest discharge from the urethra, this feature would debar his union. Proper treatment was then instituted and carried out rather more earnestly by the patient. In August there was some improvement, and he was still more anxious to get married. I suggested to him the advisability of having a microscopical examination made, and he was accordingly sent to Dr. Vissman, with instructions to take a sample of urethral discharge when he had not cleaned out the urethra for several hours, so that the discharge could be examined. This was done, and at my request Dr. Vissman made a very careful examination, and made the following report, that while there was possibly very little danger, a few gonococci were still present, and he did not think immediate marriage was advisable, as he did not consider it safe to take the risk. We then worked even more vigorously in the matter of treatment, and by the middle of September for a week or ten days there had been no discharge. The treatment was continued, and at the end of September there was still no flow. By the first of October almost a month had elapsed without the slightest sign of gonorrhea; the mucous shreds had disappeared from his urine as thoroughly as they ever disappear in any one who has had gonorrhea within five years. By a most careful examination the faintest traces of mucous shreds could be seen in the urine, but there was not the slightest evidence of discharge from the urethra, and there was nothing to examine under the microscope. I then gave it as my opinion that there was very little danger from his marrying; he was apparently in the best of health; he had been examined repeatedly for stricture, which so often follows gonorrhea, but nothing of this kind could be found, and there was no trace of the previous chronic gonorrheal infection.

The man married early in October, and, of course, pursued the usual course in those cases. November passed, and late in December he came to me, stating that his wife had been complaining, and told me the nature of her symptoms. He said that she had only been complaining for a few days. He very wisely thought an examination should be made at once, and for this purpose his wife was brought to my office. Upon examination I found that she was suffering from undoubted gonorrhea; she had urethritis, vaginitis, and considerable pain in one side, with slight pelvic peritonitis. She has been under treatment since that time; she still has vaginitis, but no urethritis, and the urine has become normal. There are still some evidences of pelvic trouble in the left side.

I cite these two cases for the following purposes: In the first place we have been taught that specific urethritis runs a certain course. It has probably not been the experience of any one here to take a case of undoubted specific (so-called) gonorrhea, such as was present in the first case, and cure it in four or five days. I remember once of having a case where I thought I was producing a remarkable result in curing a gonorrheal discharge in eleven days with resorcin. With that exception I can remember no case of specific gonorrhea that was cured inside of three to four weeks. Now, that being the case, the question is, Did this man (unfortunately we did not have the secretions examined) have a specific gonorrhea? If so, then it was cured in four or five days by remedies that have not been certain to effect a cure heretofore inside of twenty-eight days. If he did not have specific gonorrhea, how could he have given his wife gonorrhea? It is fair to assume that this man has had intercourse with his wife quite a number of times since she has gotten better, and he has not developed any form of gonorrhea.

In the second case the man had no discharge whatsoever from the urethra for several months previous to marriage, and has had none up to this time, and yet his wife developed an undoubted case of gonorrhea so far as all symptoms are concerned, because there is no form of simple vaginitis which will result in urethritis and pelvic peritonitis. I never had this woman's secretions microscopically examined.

### DISCUSSION.

Dr. GRANT: I do not speak of these cases from a specialist's standpoint, but it occurs to me that both cases Dr. Bloom describes may be easily explained. In the first case, I believe that his patient most certainly had simple urethritis, and it is entirely possible for simple urethritis to have produced this irritation in the woman. I am sure that I have seen a number of cases of urethritis which were simple in the male, certainly cases in which there were no gonococci present, perhaps in many instances there was certain irritation, the result of old strictures producing long-standing trouble, that infected the wife afterward; and while I believe, as Dr. Bloom states, that it is not possible to bring about a cure of gonorrhea in four or five days, yet I do think it possible for simple urethritis to get well in that length of time, to all appearances, and yet afterward cause vaginitis, urethritis, etc., in the female.

Referring to his second case, a great many cases of gonorrhea are supposed to be cured, while there still remains in the glands, far back in the prostatic portion of the urethra, a quantity of the gonococci, which are not in active, irritative and aggressive condition, yet they are present, and may become dislodged and pass out with the urine or with the semen in sexual intercourse, and, in the latter case, they are injected into the female vagina, and may be productive of serious trouble. I think there is almost no question about this being the course of infection in the second case reported by Dr. Bloom, and I can see no other rational explanation of the first one. I am sure Dr. Bloom will say, without hesitation, that a very large number of cases of gonorrhea are apparently cured, still there may be retained somewhere, either in the epithelial covering of the urethra or in some of the glands or pockets far back, germs able to cause gonorrhea for a very considerable length of time, even after all manifestations have apparently been cured, and this, too, without there being any particular irritation in the male, or, at least, any irritation that he is able to define as a result of the urethritis.

Dr. Cecil: We know it to be a fact that gonorrhea may exist for a long time in the genital passages of women, in the glands of Bartholini, in the cervix, in the posterior vaginal fornix, and in many places almost inaccessible, which generally are overlooked or neglected in any method of treatment that is adopted. The question as to how long gonorrhea may exist either in the male or female, and how long it may possibly be communicated, is one to which I have never been able to give any satisfactory answer.

I can hardly agree with Dr. Grant that simple vaginitis will show up in as virulent a form as indicated in the case reported by Dr. Bloom. We may have simple vaginitis occasionally, but I have never seen it involve the urethra to any extent. I have seldom seen it extend to the pelvic region and involve the tubes; in fact, I hardly think it probable that simple vaginitis will ever result in these complications. I am free to say that I cannot understand how the first case described by Dr. Bloom could have originated. The second case, especially in its relation to the time when it is advisable for persons to marry after having had gonorrhea, strikes me as being one of very great moment, because of the prevalence of gonorrhea among young men, and especially since we know the wonderful number of diseases in the female that result from so-called cured gonorrhea. I had a case in mind, at the time Dr. Bloom was speaking, of a gentleman whose wife had been suffering from some womb trouble, who had of necessity to live apart from his wife, and had indulged his fancy outside, contracting gonorrhea. He came to me for treatment, while his wife was going to another physician for treatment. I persisted with and urged upon him the necessity of complete cure before he again went to stay with his wife, and thought that I had accomplished this, and he thought so too. Every symptom had disappeared, and he went so far as to make a crucial test by going outside again and trying it on somebody else, and utterly failed in his attempt to communicate the disease. Three or four weeks after all symptoms had subsided he communicated gonorrhea to his wife.

It is a question that it seems to me ought to be settled in some definite way, if possible, because of its importance. Of course, no man wants to communicate gonorrhea to his wife, whether she be newly married or otherwise, and especially since we know that so many pelvic diseases, which require extensive operative procedure to relieve, are due to gonorrhea, it seems to me the question ought to be discussed and settled. I am sorry that I have not something definite to offer, but it seems to me that Dr. Bloom, in the cases he reports, did follow out every possible precaution that even the most exacting of us could have asked, and yet his cases went wrong. The question will come up to all of us, When can a man marry, after he has had gonorrhea, with safety? When should he be allowed to marry? The question becomes one of the greatest gravity, and should receive our careful consideration.

Dr. Bloom: My object in citing these two cases was to get the different opinions on the subject. I am fully cognizant of the experiments and tests that have been made as to the cause of gonorrhea, and, as yet, I am not satisfied and not prepared to state that the germ of Neisser is not the specific germ of gonorrhea; on the other hand, I am also not prepared to state positively that it is the essential germ which produces gonorrhea.

As regards the first case, Dr. Cecil has answered Dr. Grant exactly as I should have done. I do not believe simple urethritis could possibly produce the symptoms which were seen by one of the physicians present, who was called in consultation when peritonitis developed.

In the second case, the man married early in October, and had intercourse with his wife during October, November and a part of December before she developed any symptoms of gonorrhea, and the strangest feature is, without a single symptom developing in himself. It is natural to suppose any latent urethritis that might exist would be aggravated by sexual congress the first few weeks of marriage, and be at once communicated; I believe that is the usual history of these cases. However, in this case, nothing developed in the wife until fully two months after marriage. Some writers claim that a man may be absolutely free from all symptoms for a year, and then communicate gonorrhea. Further, do not some authorities recommend marriage for the cure of so-called gleety discharges in the male, and have not cures resulted, in some cases, without any trouble developing subsequently in the female? Yet here is a case where exactly the opposite condition exists ; there was absolutely no sign of urethral discharge for several weeks before marriage, all evidence of gonorrhea having disappeared, yet the wife became infected. We could all cite cases where a discharge was still present, the patient married, became well, and the wife was not infected. I do not believe that the gonococci can exist in any part of the urethra for a long time sufficiently active to give a specific gonorrhea to any one without causing trouble, and specific trouble at that, in the patient himself. Furthermore, if they did exist in the urethra, I believe evidences of their presence could be detected in the urine. If they existed and remained in the urethra, they could not be active and could not be made to be active, and in order to be conducted with the semen and not to flow from the urethra they would have to be present in some quantity, and serious inflammation would be likely to result therefrom.

In regard to the specificity of the bacillus of Neisser, I do not think it has been fully established, clinically at least.

Dr. Grant: In the present history of diseases of women, am I not correct in saying that there are certain authorities who maintain that no woman ever marries a man who has had gonorrhea but suffers in some way from the effect of that gonorrhea? There is abundant authority in every surgical journal of the land, and in nearly every surgical book, to support the statement I made, that gonorrhea is not by any means cured when the discharge ceases, and that in many instances the disease remains latent for a very considerable time. Therefore, if it be true, as maintained, that any woman who marries a man who has had gonorrhea invariably suffers from its effects, it would seem to me that if a man marries a woman within a period of two or three years after he has apparently recovered from an attack of gonorrhea, it is perfectly possible for the gonococci to produce such effects as Dr. Bloom has described.

The question as to when it is safe for a man to marry after having apparently recovered from an attack of gonorrhea is one of the greatest importance, and one which, in my opinion, is far from being satisfactorily settled.—*American Practitioner and News,* May 5, 1894.

---

Effect of Mercury on the Kidneys.—Welander (*Hygiea,* lvi., 2, pp. 105, 148, 1894,) has had the opportunity of observing 97 cases of syphilis before treatment with mercury and during the administration of the drug. He found that its elimination was accompanied by a greater or less irritation of the kidneys, manifested by casts in the urine. These casts increase in proportion to the length of the treatment, gradually decrease after its cessation, and generally disappear within a month or six weeks. No injury to the kidneys, either temporary or permanent, was observed.—*Universal Medical Journal.*

